# Reversible Magnesium Metal Anode Enabled by Cooperative Solvation/Surface Engineering in Carbonate Electrolytes

**DOI:** 10.1007/s40820-021-00716-1

**Published:** 2021-09-14

**Authors:** Caiyun Wang, Yao Huang, Yunhao Lu, Hongge Pan, Ben Bin Xu, Wenping Sun, Mi Yan, Yinzhu Jiang

**Affiliations:** 1 School of Materials Science and Engineering, State Key Laboratory of Clean Energy Utilization, Zhejiang University, Hangzhou, 310027 Zhejiang People’s Republic of China; 2 ZJU-Hangzhou Global Scientific and Technological Innovation Centre, Zhejiang University, Hangzhou, 311200 People’s Republic of China; 3 Institute of Science and Technology for New Energy, Xi’an Technological University, Xi’an, 710021 People’s Republic of China; 4 Mechanical and Construction Engineering, Faculty of Engineering and Environment, Northumbria University, Newcastle upon Tyne, NE1 8ST UK

**Keywords:** Rechargeable magnesium batteries, Metal anode, Solvation effect, Passivation, Carbonate electrolytes

## Abstract

Magnesium metal anode holds great potentials toward future high energy and safe rechargeable magnesium battery technology due to its divalent redox and dendrite-free nature. Electrolytes based on Lewis acid chemistry enable the reversible Mg plating/stripping, while they fail to match most cathode materials toward high-voltage magnesium batteries. Herein, reversible Mg plating/stripping is achieved in conventional carbonate electrolytes enabled by the cooperative solvation/surface engineering. Strongly electronegative Cl from the MgCl_2_ additive of electrolyte impairs the Mg…O = C interaction to reduce the Mg^2+^ desolvation barrier for accelerated redox kinetics, while the Mg^2+^-conducting polymer coating on the Mg surface ensures the facile Mg^2+^ migration and the effective isolation of electrolytes. As a result, reversible plating and stripping of Mg is demonstrated with a low overpotential of 0.7 V up to 2000 cycles. Moreover, benefitting from the wide electrochemical window of carbonate electrolytes, high-voltage (> 2.0 V) rechargeable magnesium batteries are achieved through assembling the electrode couple of Mg metal anode and Prussian blue-based cathodes. The present work provides a cooperative engineering strategy to promote the application of magnesium anode in carbonate electrolytes toward high energy rechargeable batteries.

**Supplementary Information:**

The online version contains supplementary material available at 10.1007/s40820-021-00716-1.

## Highlights

- A cooperative solvation/surface engineering approach is reported to achieve the reversible Mg plating/stripping in conventional carbonate electrolytes.
- Benefitting from the strategy, Mg^2+^ can easily overcome the reduced desolvation barrier and penetrate the Mg^2+^-conducting polymer coating, deposited on the Mg metal anode successfully, promoting the application of magnesium anode in carbonate electrolytes toward high-energy rechargeable batteries.

**Supplementary Information:**

The online version contains supplementary material available at 10.1007/s40820-021-00716-1.

## Graphical abstract

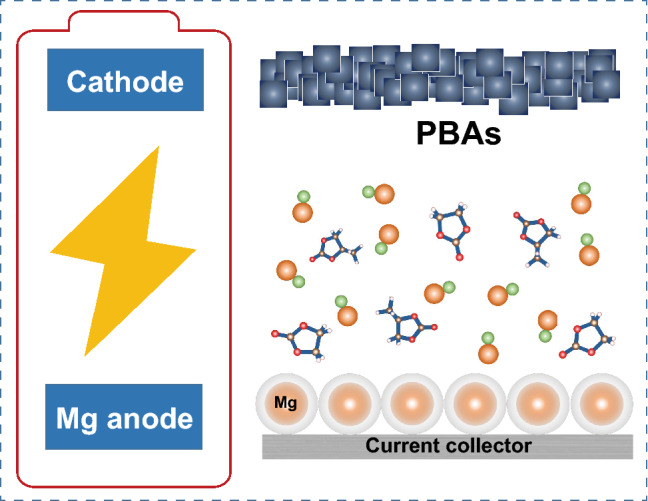

## Introduction

The last decades have witnessed a massive spurt of rechargeable lithium ion batteries (LIBs) in electrifying our modern lives ranging from portable electronics to electric vehicles [1–4]. While the global renewable energy campaign to ‘*Net Zero*’ further promotes the application of LIBs in the emerging market of energy storage [5, 6], the market also has shown persisting requirement on higher energy density and more cost-effectiveness by seeking the alternative rechargeable battery products. With this term, rechargeable magnesium batteries (RMBs) attract sustained attention due to the divalent redox and fascinating volumetric capacity of Mg anode (3832 mAh cm^−3^) [7–10]. More importantly, its resource abundance and dendrite-resistant nature enable magnesium battery as a promising and competitive sustainable and safe technology for energy storage.

The research on electrolyte solutions in the RMBs can be traced back to 1920s, when the scientists discovered Grignard regents for reversible electrochemical Mg deposition [12]. This finding opened the avenue in the pursuit of RMBs followed by the development of a series of Mg salts in ethereal solvents, classified by carbanion such as organometallics-based and hydride anions such as MgBH_4_ [13–16]. Although the reversible Mg plating/stripping has been well identified in these electrolyte solutions, they still suffer from the intrinsically poor anodic stability, the complicated synthesis procedure and the high sensitivity to air/moisture, which severely restrains the superiority of Mg batteries into full play [17]. Conventional electrolytes based on simple salts and carbonate/ether solvents have made huge success in the commercialization of LIBs, which enable the use of high-voltage cathode toward high energy batteries [18–21]. Unfortunately, they are generally considered to be incompatible with Mg metal due to the strong solvation effect and the spontaneous passivation on the anode/electrolyte interface [22–24]. In the carbonate electrolytes, the strong Mg…O = C interaction impedes the desolvation of Mg^2+^ at the interface. Even worse, the products between highly reactive Mg and aprotic organic solutions exhibit nonconducting characteristic for both Mg^2+^ and electrons, hindering the subsequent Mg plating/stripping [25].

To enable the compatibility of conventional electrolytes in Mg-based batteries, multiple attempts have been carried out including building artificial interphase, using Mg-based alloy anode, and adding electrolyte additives [26, 27]. Ban et al. engineered an artificial Mg^2+^-conducting interphase on the Mg anode surface to achieve highly reversible Mg chemistry in carbonate electrolyte [28]. Wang et al*.* employed iodine additive in electrolyte to construct ion-conducting surface layer on Mg anode [29]. Luo et al. proposed to modify Mg metal anode with Sn-based artificial layer for rechargeable magnesium battery in conventional electrolyte [30]. Unfortunately, the quest remains unsettled on resolving the multiple challenges related to Mg metal anode, including surface passivation and desolvation barrier in conventional electrolytes.

Herein, we demonstrate reversible Mg plating/stripping in conventional carbonate electrolyte through a cooperative strategy to engineer the desolvation barrier and the Mg^2+^-conducting surface layer. The Mg^2+^-conducting and electron-insulating feature of polymer coating on Mg anode enables a facile Mg^2+^ transport and subsequent Mg redox reaction, which isolates the electrolyte to avoid parasitic reactions and surface passivation. On the other hand, strongly electronegative Cl of additive MgCl_2_ weakens the interaction of Mg…O = C in the electrolyte, reducing the Mg^2+^ desolvation barrier for accelerated reaction kinetics. Accordingly, a reversible Mg anode/carbonate electrolyte system is achieved with stable Mg plating/stripping at an overpotential of 0.7 V for over 2000 cycles. By coupling with Prussian blue analogs (PBAs) cathodes, magnesium full cells in carbonate electrolytes achieve high-voltage charge/discharge toward high energy rechargeable magnesium batteries.

## Experimental Section

### Fabrication of Coated Mg Electrodes

The bare-Mg anode was made of Mg powders, super P and polyvinylidene fluoride (PVDF) with a weight ratio of 8:1:1. After these powders were uniformly mixed in NMP, the slurry was equably coated on stainless steel foil, the electrode was dried naturally in the argon-filled glove box. The coated Mg anode consisted of 70 wt% Mg powders, 10 wt% super P, 10 wt% polyethylene oxide (PEO) and 10 wt% Mg(TFSI)_2_. PEO and Mg(TFSI)_2_ were dissolved into a moderate amount of acetonitrile in advance. After stirring about 5 h, Mg powders and super P were sequentially added to the solution and continue to mix them evenly into a slurry. The coated Mg anode was prepared by scraping the uniform slurry on stainless steel foil. The above operations are carried out in the argon-filled glove box.

### Preparation of Electrolytes

The MgCl_2_/Mg(TFSI)_2_ solutions were prepared by adding 0.1 M MgCl_2_ and 0.5 M Mg(TFSI)_2_ into EC/PC (1:1, volume ratio) and stirred until dissolved. After using molecular sieve to remove water for over 24 h, the supernatant was taken for the subsequent battery assembling.

### Synthesis of Cathode Materials

The coprecipitation method was applied to obtain NiHCF cathode. 0.3 mol Na_4_Fe(CN)_6_·10H_2_O was dissolved in 1 L deionized water, marked as solution A. 0.3 mol NiCl_4_·4H_2_O and 1.2 mol sodium citrate were dissolved in 1 L deionized water and formed solution B. Solution B was added into solution A (25 ℃) by a peristaltic pump at a rate of 2 mL min^−1^ under magnetic stirring and then aged for 1 h to obtain a suspension. The obtained NiHCF powders were collected after centrifugation and dried in vacuum at 120 ℃ for 24 h. MnHCF was synthesized by the EDTA-assisted co-precipitation method. 0.01 mol EDTA-MnNa and 0.01 mol Na_4_Fe(CN)_6_·10H_2_O were dissolved in 200 mL deionized water, marked as solution A. 0.02 mol citric acid was dissolved in 100 mL deionized water and formed solution B. Solution B was added into solution A (25 °C) by a peristaltic pump at a rate of 0.5 mL min^−1^ under magnetic stirring and then aged for 6 h to obtain a suspension. The obtained MnHCF powders were collected after centrifugation and dried in vacuum at 120 °C for 24 h.

### Materials Characterization

The structural characterization of the materials were carried out by X-ray diffraction (XRD, Bruker D8 Advance, Germany) with Co Kα radiation (*λ* = 1.7902 Å) at 35 kV, 28 mA. The morphologies of samples were characterized by scanning electron microscopy (SEM, HITACHI SU8010) and transmission electron microscope (TEM, HITACHI HT7700). FT-IR spectra of the interphase were obtained using a FT-IR spectrometer (Nicolet 5700) and an ATR unit running from 400 to 4000 cm^−1^. All the Raman spectra were recorded by a laser confocal Raman spectrometer (LabRAM HR Evolution) with the resolution of 0.65 cm^−1^. The spectrum region from 1900 to 1700 cm^−1^ was fitted with Lorentzian line shapes.

### Electrochemical Measurements

The ionic conductivity of the coating film was measured by a cell configuration of the Mg^2+^-conductive interphase sandwiched between two stainless steel disks, as shown in Fig. S1. The ionic conductivity can be calculated by the following formula:

1 $$\sigma =\frac{d}{R\bullet \mathrm{S}}$$

where *d* is the thickness of the Mg^2+^-conductive interphase (0.24 mm) and *S* is the area of the stainless steel disks. *R* is determined by the Nyquist plot. CR2025 coin cells were assembled in the glove box with the use of Whatman glass fiber membranes for subsequent electrochemical investigations. The symmetrical battery consists of two completely identical electrodes. For full cells, cathode materials, super P and PVDF in the weight ratio of 7:2:1 in NMP solvent were mixed. After the slurry was uniformly coated on the Ti foils (0.01-mm-thick), the electrodes were dried in a vacuum drying oven at 120 °C for 12 h. The average mass loading of active material was around 1.0 mg cm^−2^. CR2025 coin cells were assembled in an Ar-filled glove box with H_2_O and O_2_ contents less than 1 ppm. Each cell was filled with 80 ~ 120 μL electrolytes before sealed. The cathode materials were desodiated in Na-ion batteries before assembled for Mg batteries. The battery system (Neware BTS-5) was used to carry out galvanostatic tests of the cells. Electrochemical impedance spectroscopy (EIS) was examined in the frequency range from 1 MHz to 1 Hz with an amplitude of 5 mV by a CHI660C electrochemical workstation.

### Calculation Method

Density functional theory (DFT) calculation was used in the VASP, a first-principles calculation code with high precision using the PAW method. We adopted PBE as the term exchange correlation with a cutoff energy of 400 eV and all calculations performed nonmagnetically. Grimme’s method (DFT-D3) was employed to incorporate the effects of van der Waals interactions. For the interaction between two molecules, we have established a 30 × 30 × 30 vacuum layer, the calculation was based on a mesh of 1 × 1 × 1 in k-point grid. All atoms were full relaxed until the force on them was less than 0.01 eV Å^–1^. The energy of interaction between groups (ΔE) was calculated by the formula:

2 $$\Delta {\text{E}} = \Delta E_{{{\text{total}}}} - E_{A} - E_{B}$$

where *E*_*total*_ was the total energy of the systems and *E*_A_ was the energy of the optimized A atomic group.

## Results and Discussion

### Principle and Effects of the Cooperative Strategy

**Fig. 1:**
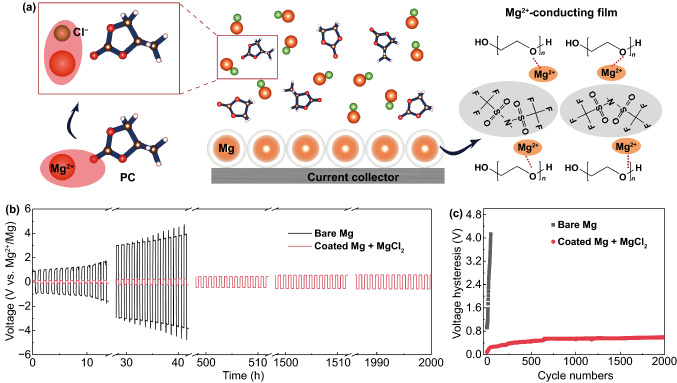
**a** Schematic diagram of cooperative solvation/surface engineering. **b** Voltage responses of symmetric Mg batteries with and without collaborative regulation in 0.5 M Mg(TFSI)_2_ electrolyte systems at a current density of 0.01 mA cm^−2^ where each deposition/stripping cycle lasts for a half hour. The cell made with pristine Mg electrodes shows huge overpotential at the beginning and fails after 30 cycles, whereas the cell made with the cooperative engineering of solvation and interface performs prolonged cycles in carbonate-based electrolytes. The reversibility is conspicuous in the latter as proven by up to 2,000 cycles. **c** Voltage hysteresis versus cycle numbers for symmetric Mg electrodes. Lower voltage hysteresis is observed on modulated Mg electrode and electrolyte

The cooperative strategy on regulating the solvation structure and the surface polymer coating is schematically illustrated in Fig. 1a. Mg(TFSI)_2_ in ethylene carbonate (EC)/propylene carbonate (PC) (1:1, volume ratio) is chosen as the model electrolyte solution in this study. Such carbonate electrolytes with much wider voltage window compared with commonly used electrolytes (i.e., APC ((PhMgCl)_2_-AlCl_3_/THF), Fig. S2) exhibit extraordinary potential toward high-voltage Mg batteries. However, Mg^2+^ interacts tightly with carbonyl group in carbonate electrolyte, in which part of the electrons are transferred from the solvent molecules to Mg^2+^. Each Mg^2+^ is surrounded by 6 C = O ligands to form Mg(PC)_6_^2+^ and Mg(EC)_6_^2+^, hindering the Mg^2+^ release from the solvation cage and subsequent Mg deposition process [24]. To reduce Mg^2+^ solvation energy in the conventional carbonate electrolyte solutions, strongly electronegative Cl element through the addition of MgCl_2_ in electrolyte is selected to grab electron clouds around Mg [31]. As a result, the strong Mg…O = C interaction in solvents could be weakened to accelerate the Mg^2+^ release for Mg plating. On the other hand, the poor reduction stability makes carbonate electrolytes extremely reactive with Mg anode, forming an ion-insulating passivation layer on the Mg surface that leads to the failure of Mg metal anode [25].

We further engineer a polymer based Mg^2+^-conducting coating on Mg metal particles to structurally block the direct contact and minimize the parasitic reactions between electrolyte and Mg metal, enabling reversible Mg plating/stripping. The polymer coating consists of polyethylene oxide (PEO) and Mg(TFSI)_2_, in which the lone pair of oxygen of the PEO segment will be coordinated with Mg^2+^ by Coulombic interaction [32, 33]. Meanwhile, the enormous TFSI^−^ anions can effectively degrade the crystallinity of PEO for the accelerated movement of polymer segments to achieve enhanced Mg^2+^ migration [34]. Symmetric cell with controlled Mg electrode in exhibits very short cycle life along with ever-increasing overpotential, such cooperative strategy contributes to the reversible Mg plating/stripping up to 2000 cycles with a much reduced and stable overpotential of 0.7 V in the carbonate electrolyte (Fig. 1b, c).

### Characterization of the Polymer Coating

**Fig. 2:**
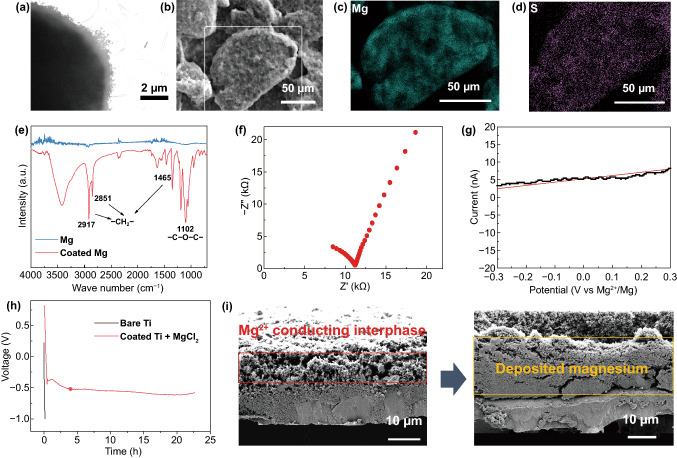
**a** TEM imaging of the Mg^2+^-conductive interphase. **b** SEM imaging of the coated Mg particle and the EDS mapping area is indicated by the white rectangular box. **c, d** EDS elemental mapping of Mg and S. **e** IR spectrum of Mg electrode with and without the Mg^2+^-conductive interphase. **f** Impedance spectra and **g** linear sweep voltammogram of the Mg^2+^-conductive interphase. The Mg^2+^-conductive interphase is sandwiched between two stainless steel disks, which act as ion-blocking electrodes.** h** Voltage profiles of Mg deposition on Ti current collector with and without the Mg^2+^-conductive interphase in 0.5 M Mg(TFSI)_2_ electrolyte at a current density of 0.01 mA cm^−2^. **i** Cross-sectional SEM image on pristine-coated Ti electrode and the same electrode after 22 h of Mg deposition. The area of Mg^2+^-conductive interphase is indicated by the red rectangular box, while the area of deposited Mg is indicated by the yellow rectangular box

The surface polymer coating was first characterized by transmission electron microscopy (TEM) and energy-dispersive X-ray spectroscopy (EDS) elemental mapping as shown in Fig. 2a–d. Around 500-nm-thick polymer layer has been uniformly coated on Mg particles with homogeneous distribution of Mg and S elements from Mg(TFSI)_2_, indicating the success in the coating process. The existence of PEO is verified by infrared spectrum (IR) as shown in Fig. 2e, recorded with C–O–C and CH_2_ vibration peaks of PEO [35]. As compared to the strong crystalline peaks of PEO in the XRD pattern, PEO peaks become undetectable for the conductive coating (Fig. S3). Such degradation in crystallinity of PEO can be attributed to the incorporation of the TFSI^−^ anions, which is expected for the enhancement of Mg^2+^ conduction [36].

Electrochemical impedance spectroscopy (EIS) and linear sweep voltammetry (LSV) measurements with the sandwich cell configuration were taken to evaluate the ionic/electronic conductivity of the polymer coating at room temperature (Fig. 2f–g) [37]. As a result, an ionic conductivity of 1.1 × 10^–6^ S cm^−1^ is obtained, which is quite high in the consideration of the divalent Mg^2+^ that possesses high Coulombic force [38]. The high ionic conductivity and good mechanic flexibility of polymer electrolyte coating ensure the facile Mg^2+^ transport with good accommodation for reversible Mg plating/stripping. Meanwhile, the electronic conductivity of such polymer coating is recorded as 1.2 × 10^–10^ S cm^−1^, four orders of magnitude lower than its ionic conductivity, rendering the long-term and stable cycling due to the inhibition of the parasitic reactions of electrolyte and the formation of passivation layer. To identify the coating effect on the electrochemical behavior of Mg deposition, Ti was used to study the Mg plating behaviors on the polymer coating. Galvanostatic test was performed with both bare Ti and coated Ti as working electrodes with Mg counter one. As expected, the deposition process is extended to 22 h in contrast to the deposition failure of bare Ti (Fig. 2h-i). Such polymer coating can be extended to other electrolytes such as APC and DME (1,2-dimethoxyethane), leading to a reduced overpotential and stable Mg deposition process (Fig. S4).

### Effect of the MgCl_2_ Additive

**Fig. 3:**
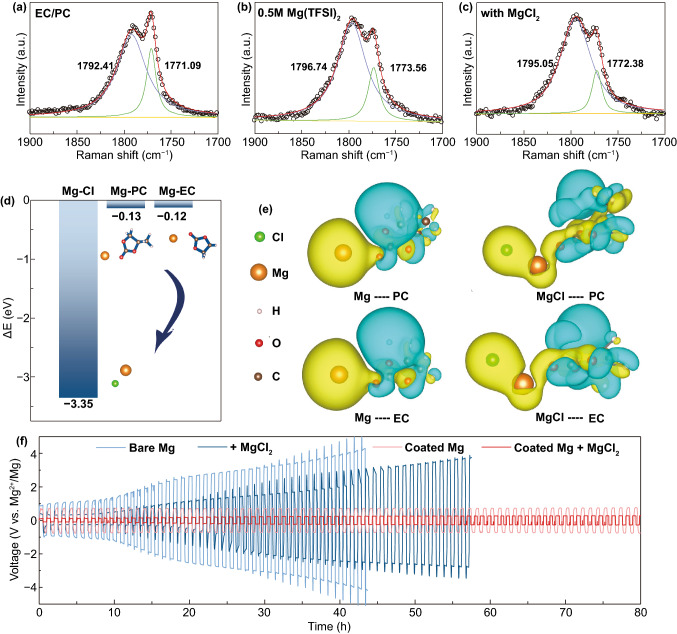
FT-Raman spectra of the following solution: **a** EC/PC (1:1, volume ratio), **b** 0.5 M Mg(TFSI)_2_ in EC/PC (1:1, volume ratio), **c** MgCl_2_/Mg(TFSI)_2_ electrolyte. **d** The calculated interaction energy of Mg-Cl, Mg-PC and Mg-EC. **e** Differential charge density plots for the group of Mg and solvent molecules with or without Cl. The yellow color shows the aggregation of electron cloud while blue color indicating the loss of electron cloud. **f** Voltage responses of symmetric Mg batteries with and without Mg^2+^-conductive interphase and MgCl_2_ additive

To further assess the effect from the MgCl_2_ additive, the chosen electrolyte solutions were analyzed by Raman spectroscopy as shown in Fig. 3a–c. In the electrolytes, the two peaks assigned from 1900 to 1700 cm^−1^ are associated with the stretching vibration of the carbonyl group [39]. As reported, the carbonyl stretching vibration can be split to two peaks, symmetrical vibration (1771.09 cm^−1^) and antisymmetrical vibration (1792.41 cm^−1^) [40]. These two peaks shift to high frequency (1773.56 and 1796.74 cm^−1^) when Mg(TFSI)_2_ is added, illustrating the strong Mg…O = C interaction [41]. Upon further adding MgCl_2_ additive, the two peaks shift back to low frequency (1772.38 and 1795.05 cm^−1^), implying the interaction of Mg…O = C has been weakened. To study the feasibility of MgCl_2_ additive in regulating the solvation structure, DFT calculations were carried out to compare the interaction energy among Mg…Cl, Mg…PC, and Mg…EC systems (Fig. 3d) [42]. The detail solvation structures of Mg with EC and PC are shown in Fig. S5. The interaction energy of Mg…Cl (-3.35 eV) is predicted to be greatly reduced compared with Mg…PC (−0.13 eV) and Mg…EC (−0.12 eV), suggesting that Mg is inclined to interact with Cl rather than solvent molecules. The differential charge density diagrams visually present the charge transfer during the interaction (Fig. 3e) [43]. Without adding MgCl_2_, the electrons are aggregated around Mg while the losing of electrons happens in the vicinity of the solvents, confirming the strong interaction of Mg…O = C. When adding MgCl_2_, there is no obvious electron aggregating around Mg and the electrons are attracted to Cl, indicating the weakened interaction of Mg…PC and Mg…EC by strongly electronegative Cl.

Galvanostatic tests on different symmetric cells were carried out to identify the role of the cooperative engineering strategy, as shown in Fig. 3f. In contrast to bare Mg in bare carbonate electrolyte, the addition of MgCl_2_ can greatly reduce the overpotential of Mg chemistry especially in the initial cycles but become invalidate in the subsequent cycles due to the passivation caused by the decomposition of the electrolyte. On the other hand, engineering the Mg^2+^-conducting polymer coating on Mg metal anode can elongate and stabilize the cycling with a still relatively large overpotential. Combined with the above cooperative strategy including the solvation and interface regulation strategy, we eventually obtained a reversible Mg anode/electrolyte system in carbonate electrolytes with a low overpotential. The modified cells exhibit a decreased resistance in EIS test, confirming the accelerated ion migration on the interface (Fig. S6). The improved kinetics originated from the cooperative solvation/interface engineering is further verified by galvanostatic cycling test of symmetric cells, which shows the reduced overpotential particularly at high current density (Fig. S7).

### Full Cells with PBAs

**Fig. 4:**
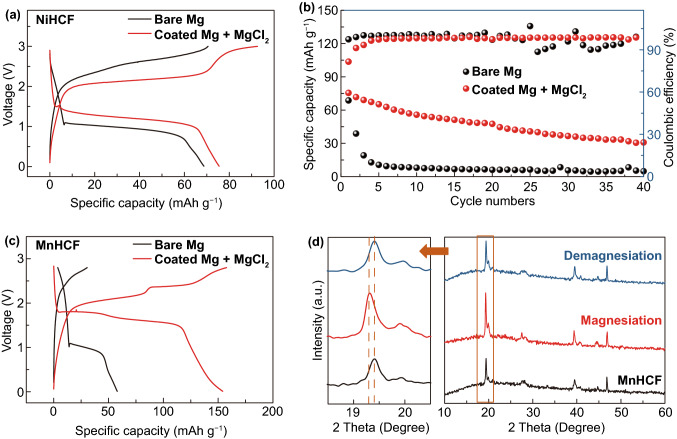
Electrochemical performance of full cells: **a** charge–discharge curves and **b** cycling performance at 0.1C for NiHCF/bare-Mg and NiHCF/Coated Mg + MgCl_2_ full cells. **c** charge–discharge curves at 0.1C for MnHCF/bare-Mg and MnHCF/coated Mg + MgCl_2_ full cells. **d** XRD patterns for MnHCF at the different states of charge

We then envisage the potential of cooperative strategy in practical application, by assembling the full cells with Prussian blue analogs (PBAs), also called as HCFs, as cathode materials. Although PBAs have been well known for the capability of magnesium storage, the prototype cells with PBAs cathode and Mg metal anode have never been reported elsewhere, possibly due to the limitation in the voltage window of the state-of-the-art electrolytes [44–46]. The morphologies and phase characterizations of NiHCF and MnHCF are displayed in Fig. S8. As displayed in Fig. 4, the capacity of NiHCF/bare-Mg decays nearly 100% after five cycles at 8 mA g^−1^ in bare carbonate electrolyte. In contrast, the cycling stability of the NiHCF/coated-Mg in the MgCl_2_-added carbonate electrolyte was greatly promoted with a much decreased overpotential. The subsequent capacity decay might be attributed to the unavoidable water impurities in PBAs [47]. MnHCF possesses higher voltage platforms and higher theoretical capacity derived from its two-electron redox [48]. The MnHCF/coated-Mg in the MgCl_2_-added carbonate electrolyte achieves improved capacity (154 mAh g^−1^) and reduced overpotential compared with MnHCF/bare-Mg cell. To further prove the Mg^2+^ extraction and insertion behavior in MnHCF, *ex situ* XRD analysis was carried out to investigate the structural evolution of MnHCF with variation in the charge/discharge states. The primary (200) peak shifts to lower angle after magnesiation, indicating that inter-planar spacing is increased accompanied by the Mg^2+^ insertion. Upon demagnesiation, (200) peak moves back to its original angle demonstrating the reversible extraction of Mg^2+^. The successful demonstration of the cooperative strategy provides a comprehensive perspective for practical use of high-voltage cathode in rechargeable Mg battery.

## Conclusions

In summary, we report a cooperative engineering strategy of solvation and surface to resolve the dilemma faced by Mg batteries in carbonate electrolytes. The polymeric Mg^2+^-conducting coating provides tunnels for Mg^2+^ migration and facilitates the following deposition processes, while its electronic insulation nature prevents the reduction of electrolytes, ensuring the good reversibility of Mg plating/stripping. In addition, Cl-contained electrolyte additive is capable of capturing electrons from the solvent molecular and accelerating the release of Mg^2+^ from the solvated structure. Consequently, reversible Mg plating/stripping with low overpotential and long-term stale cycling is achieved in carbonate electrolytes. Benefitting from such cooperative approach, we also construct full cell by using PBAs as cathode for the first time, which exhibit superior electrochemical performance, demonstrating the potential of high-voltage Mg batteries based on carbonate electrolytes. This reversible Mg anode/electrolyte system enabled by the described cooperative engineering strategy has shown great promises to build high-voltage cathodes toward high energy magnesium rechargeable batteries.

## Supplementary Information

Below is the link to the electronic supplementary material.

Supplementary file1 (PDF 731 kb)
