# Supplementary material for: Reversible Magnesium Metal Anode Enabled by Cooperative Solvation/Surface Engineering in Carbonate Electrolytes
Source: Nanomicro Lett. 2021 Sep 14;13:195. doi: 10.1007/s40820-021-00716-1 (PMC8440703; doi:10.1007/s40820-021-00716-1)
Supplement: Supplementary file 1 — Supplementary file1 (PDF 731 kb) [file 40820_2021_716_MOESM1_ESM.pdf]

Supporting information for

Reversible Magnesium Metal Anode Enabled by Cooperative Solvation/Surface Engineering in Carbonate Electrolytes

Caiyun Wang[1, 2], Yao Huang[1, 2], Yunhao Lu[1], Hongge Pan[1, 3], Ben Bin Xu[4], Wenping Sun[1], Mi Yan[1], Yinzhu Jiang[1, 2, *]

[1]School of Materials Science and Engineering, State Key Laboratory of Clean Energy Utilization, Zhejiang University, Hangzhou, Zhejiang 310027, P. R. China

[2]ZJU-Hangzhou Global Scientific and Technological Innovation Centre, Zhejiang University, Hangzhou, 311200, P. R. China

[3]Institute of Science and Technology for New Energy, Xi'an Technological University, Xi'an 710021 P. R. China

[4]Mechanical and Construction Engineering, Faculty of Engineering and Environment, Northumbria University, Newcastle upon Tyne NE1 8ST, UK

*Corresponding author. E-mail: yzjiang@zju.edu.cn (Yinzhu Jiang)

Supplementary Figures

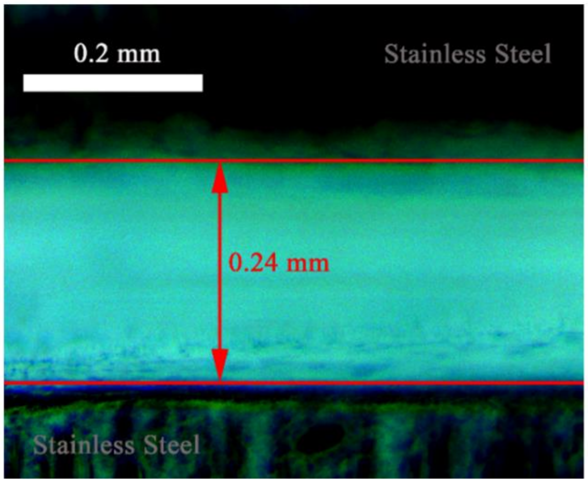

Fig. S1 Picture of the Mg^{2+}-conductive interphase under an optical microscope. The interphase sandwiched between two stainless-steel disks has a thickness of 0.24 mm.

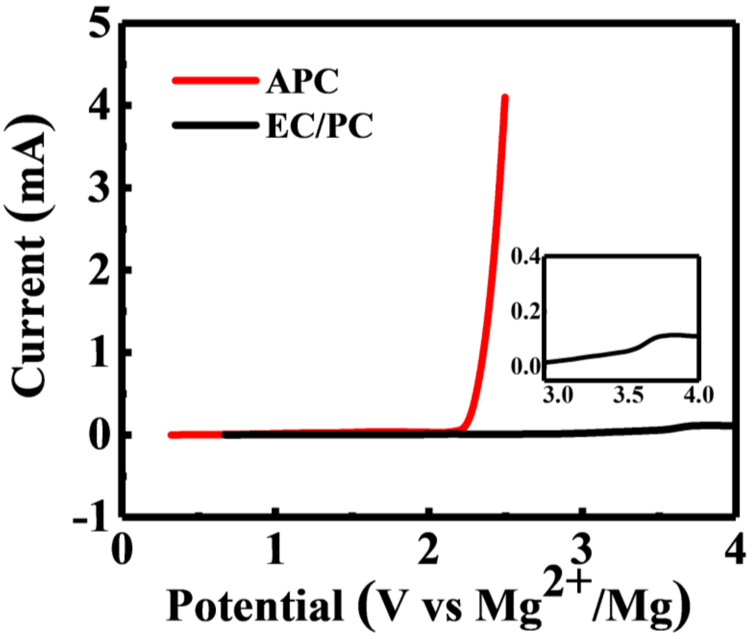

Fig. S2 LSV curve in APC and EC/PC electrolytes with Mg anode. APC electrolyte decomposes at 2.2 V, while EC/PC electrolyte can remain stable within 3.5 V.

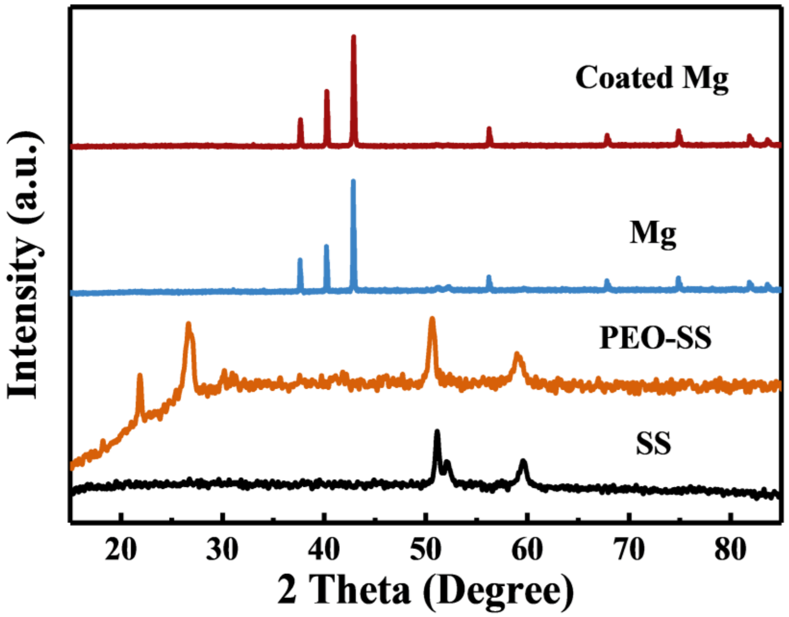

Fig. S3 XRD patterns of conducting film coated Mg, bare Mg, PEO coated stainless steel and stainless steel (SS). PEO peaks become undetectable for the conductive coating on Mg anode, which is caused by the destruction effect of crystallinity by $TFSI^-$ anions.

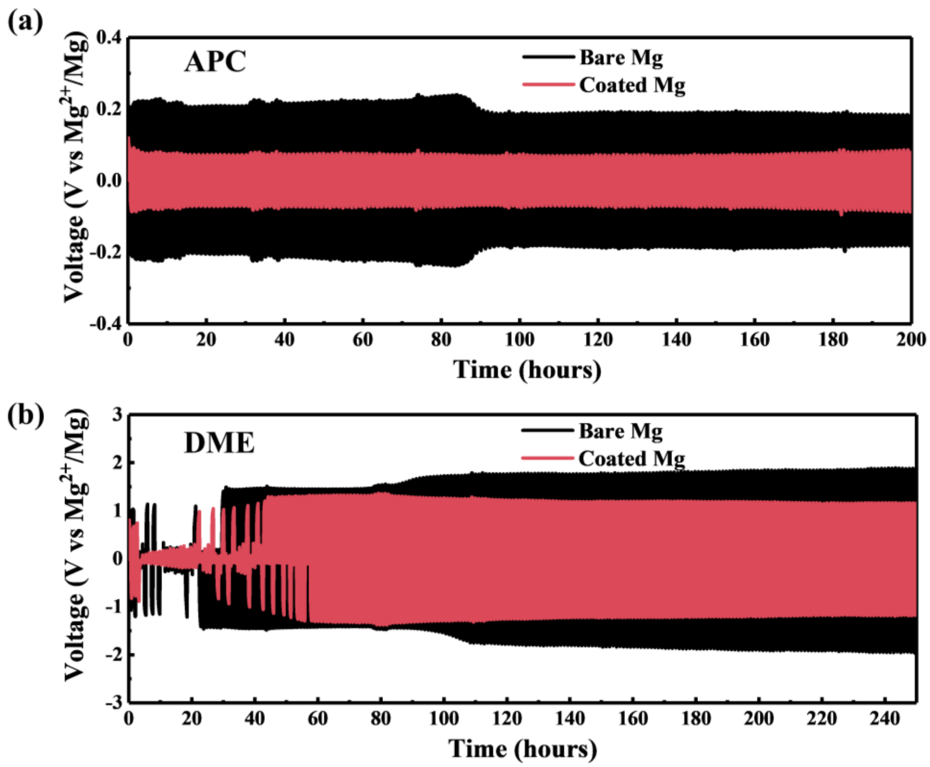

Fig. S4 Voltage responses of symmetric Mg batteries with and without the Mg^{2+}-conductive interphase in **a** APC electrolyte and **b** 0.5 M $Mg(TFSI)_2$ in DME at a current density of 0.01 mA cm^{-2} where each deposition/stripping cycle lasts for a half hour

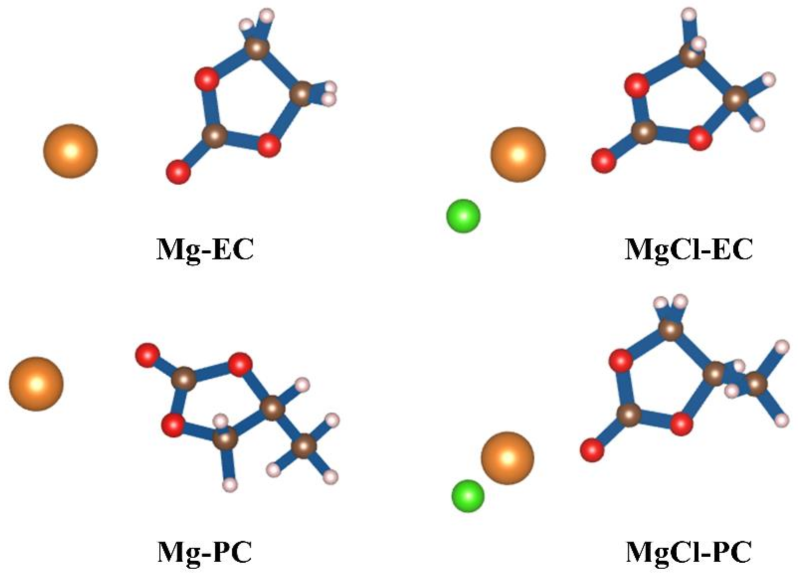

Fig. S5 Optimized solvation structures. The different colors represent different atoms (Orange-Mg, Green-Cl, Red-O, Brown-C, White-H).

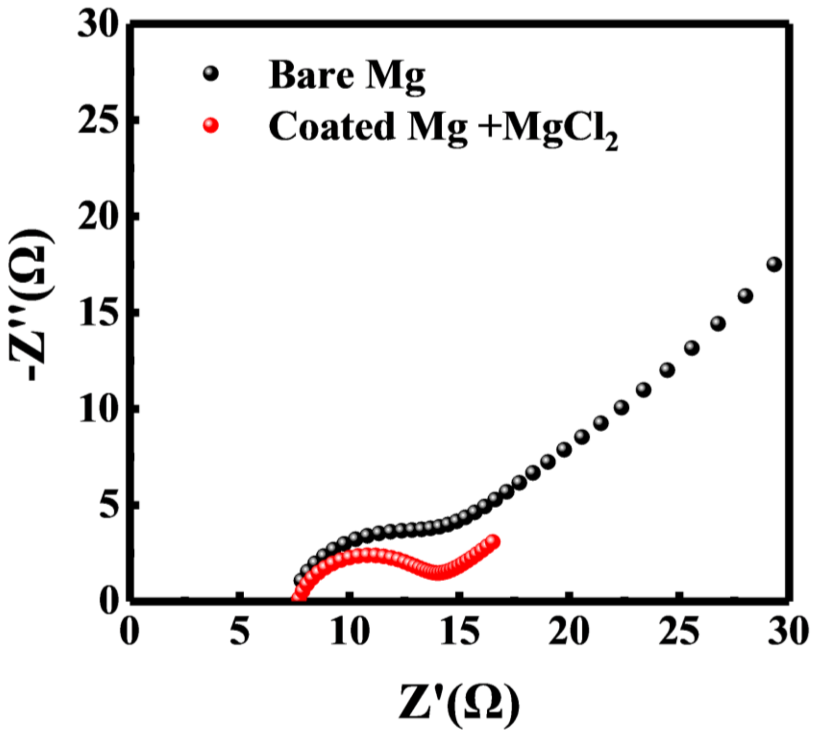

Fig. S6 Electrochemical impedance spectroscopies for cells before and after collaborative regulation

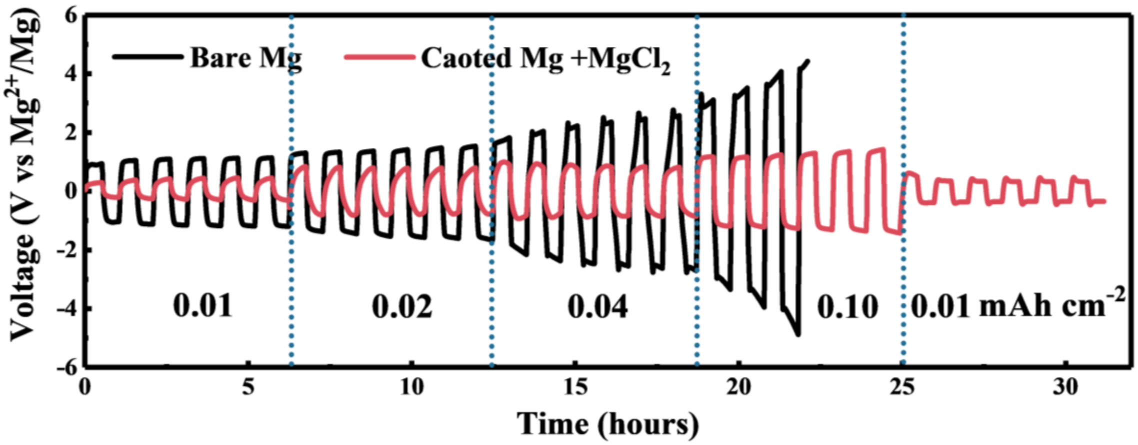

Fig. S7 Galvanostatic cycling test of symmetric cells at various current density before and after cooperative solvation/interface engineering in the carbonate electrolyte

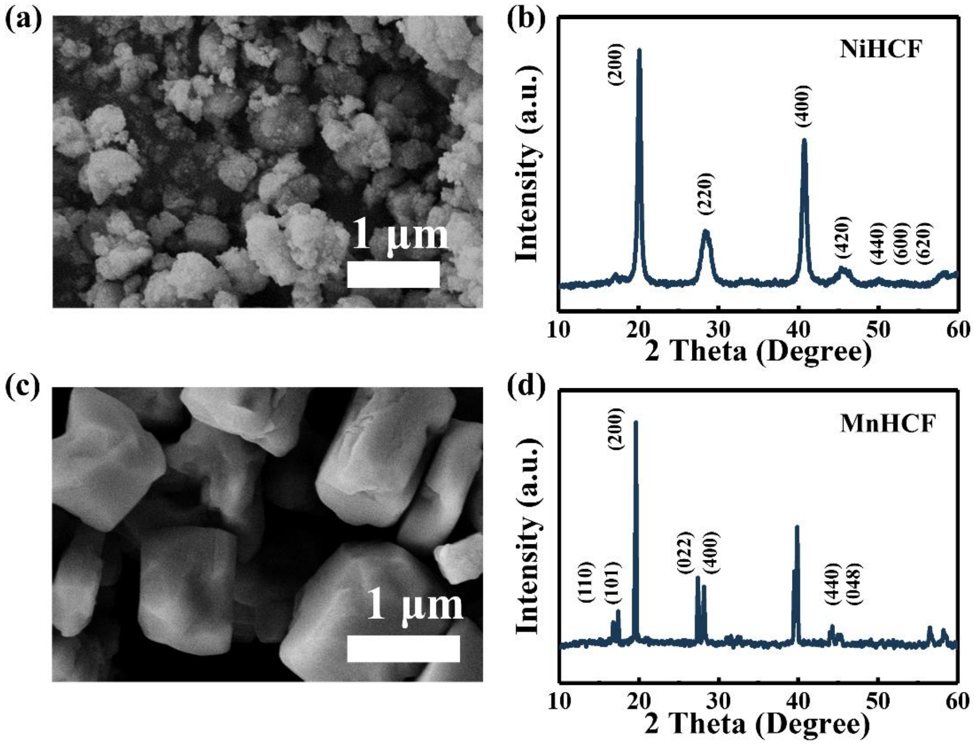

Fig. S8 (**a**) SEM image and (**b**)XRD pattern of NiHCF. (**c**) SEM image and (**d**)XRD pattern of MnHCF
